# First-principles study on the elastic, electronic and optical properties of all-inorganic halide perovskite solid solutions of CsPb(Br_1−*x*_Cl_*x*_)_3_ within the virtual crystal approximation†

**DOI:** 10.1039/d2ra01084d

**Published:** 2022-03-29

**Authors:** Un-Hyok Ko, Jun-Hyok Ri, Jong-Hyok Jang, Chol-Hyok Ri, Un-Gi Jong, Chol-Jun Yu

**Affiliations:** Chair of Computational Materials Design (CMD), Faculty of Materials Science, Kim Il Sung University PO Box 76 Pyongyang Democratic People’s Republic of Korea cj.yu@ryongnamsan.edu.kp

## Abstract

All-inorganic halide perovskites have drawn significant attention for optoelectronic applications such as solar cells and light-emitting diodes due to their excellent optoelectronic properties and high stabilities. In this work, we report a systematic study on the material properties of all-inorganic bromide and chloride perovskite solid solutions, CsPb(Br_1−*x*_Cl_*x*_)_3_, varying the Cl content *x* from 0 to 1 with an interval of 0.1 by applying the first-principles method within the virtual crystal approximation. The lattice constants of the cubic phase are shown to follow the linear function of mixing ratio *x*, verifying that Vegard’s law is satisfied and the pseudopotentials of the virtual atoms are reliable. We calculate the band structures with the HSE06 hybrid functional with and without spin–orbit coupling, yielding band gaps in good agreement with experimental results, and find that the band gap increases along the quadratic function of the Cl content *x*. With increasing Cl content *x*, the elastic constants and moduli increase linearly, the effective mass of the electron and hole increase, while mobilities decrease linearly, the static dielectric constant decreases linearly, and exciton binding energy increases quadratically. We calculate the photo-absorption coefficients and reflectivity, predicting the absorption peaks shift to the ultraviolet region from bromide to chloride.

## Abstract 2

We investigate the variation of structural, elastic, electronic, and optical properties of all-inorganic bromide and chloride perovskite solid solutions of CsPb(Br_1−*x*_Cl_*x*_)_3_ using first-principles calculations within the virtual crystal approximation.

## Graphical abstract

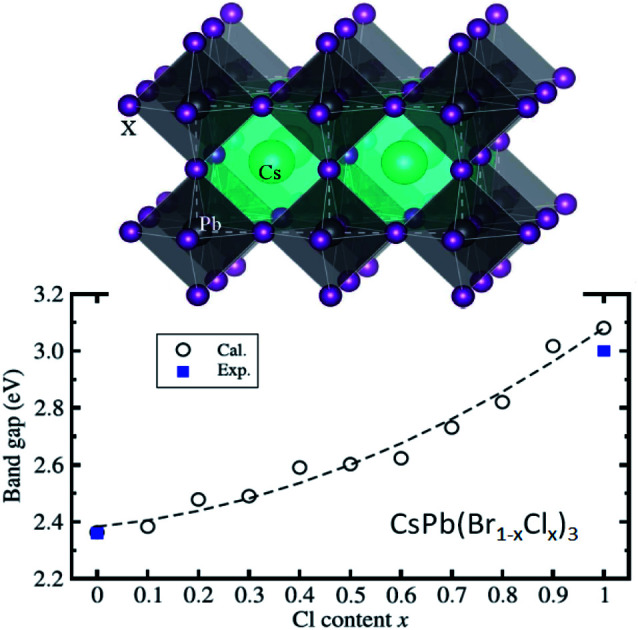

## 1 Introduction

Recently, all-inorganic halide perovskites have attracted broad interest due to their fascinating optoelectronic properties together with improved material stability,^1,2^ and significant advances in stability have already been achieved for mixed organic–inorganic hybrid halide perovskites.^3–5^ In fact, this emerging class of materials has shown promising potential in widespread optoelectronic applications such as solar cells,^6,7^ light-emitting diodes,^8–10^ photodetectors, and lasing devices.^11^ Among these, lead-based cesium halide perovskites with chemical formula CsPbX_3_ (X = I, Br, Cl) have been studied most widely for advancing perovskite solar cells (PSCs) and perovskite light-emitting diodes (PLEDs). Through the numerous experimental work, PSCs based on CsPbI_3_,^12–14^ CsPbBr_3_ (ref. 15–17) and CsPb(I_*x*_Br_1−*x*_)_3_ (ref. 18–21) were found to exhibit relatively high power conversion efficiencies of over ∼21%, and enhanced stability; while CsPbCl_3_ has proved to be useful for PLEDs rather than PSCs due to its wider band gap.

PLEDs are expected to be promising for next generation display and lighting.^8^ It has been demonstrated that the composition dependent photoluminescence (PL) of CsPbX_3_ covers the whole visible spectral range of 410–700 nm with a narrow line width of 12–42 nm and a PL quantum yield close to unity.^22,23^ In fact, CsPbI_3_ was found to exhibit red luminescence with a wavelength of ∼650 nm,^24^ CsPbBr_3_ can emit blue and green light,^24–27^ and CsPbCl_3_ shows a purple light emission.^28,29^ Moreover, it is particularly interesting to tune the color of emitting light by making solid solutions: CsPb(Br_1−*x*_Cl_*x*_)_3_ for a wavelength range from 420 to 480 nm,^28^ CsPb(I_1−*x*_Br_*x*_)_3_ for light from 520–630 nm,^23^ CsPb(I_1−*x*_Cl_*x*_)_3_ for a broader range of PL spectra.^29^ Using CsPbBr_3_ quantum dots (QDs) caste on a blue LED chip, a bright green LED was fabricated, with the PL peak and full-width at half-maximum to be tunable with a size and concentration of QDs.^25,26^ In addition, the CsPbBr_3_ QDs were made in composites with mesoporous silica, and the resultant nanocomposites exhibited excellent luminescence performance.^27^

However, developing practical PLEDs is still challenging due to the relatively short-term stability of CsPbX_3_, low external quantum efficiency of the device related with its poor morphology, and relatively high cost of fabrication. For instance, the external quantum efficiencies of green and blue PLEDs based on CsPbBr_3_ were reported to be very low such as 0.09% and 0.1% due to unbalanced charge injection.^30,31^ In particular, the cubic phase CsPbI_3_, which is the major PL-active phase, has been found to be stable only above 315 °C and readily converts to an exclusively PL-inactive yellow phase at room temperature.^32–34^ To increase the stability, anion exchange or formation of solid solutions between the different halides was in general adopted. This also provides potential for making white PLEDs with a spectrum close to sunlight by optimizing their composition.^35^ It was found that halide anion exchange in CsPbX_3_ is remarkably fast, which is beneficial and necessary for enhancing stability, promoting facile synthesis, and in particular for ensuring color tunability in PLED applications.^22,23,36^ In exchanging and mixing halide anions, Cl and I were reported to be restricted by an unfavorable lattice mismatch,^22,29^ whereas Br–I and Br–Cl are expected to be suitable for the formation of solid solutions.

There are several first-principles studies on halide perovskite solid solutions, including all-inorganic compounds^37–41^ and organic–inorganic hybrid ones,^42,43^ using the supercell method and the efficient virtual crystal approximation (VCA) approach^44^ within a density functional theory (DFT) framework. In particular, Zhou *et al.*^37^ and Ghaithan *et al.*^38^ reported the structural, electronic and optical properties of mixed halide perovskites CsPb(Br_1−*x*_Cl_*x*_)_3_ using the supercell modeling. In this work, we investigate the elastic and optoelectronic properties of Pb-based cesium bromide and chloride perovskite solid solutions of CsPb(Br_1−*x*_Cl_*x*_)_3_, gradually increasing the Cl content *x* from 0 to 1 by applying the first principles VCA approach.^44^ Our work focuses on clarifying the potential of these solid solutions toward efficient and stable PLED applications.

## 2 Computational methods

We performed the DFT calculations using the pseudopotential plane wave method as implemented in the Quantum ESPRESSO (QE, version 6.2.0) ^45^ and ABINIT (version 8.8.4) ^46,47^ packages. For all atomic species, we constructed the Troullier–Martins type norm-conserving pseudopotentials^48^ with valence electron configurations of Cs-6s^1^6p^0^, Pb-4f^14^5d^10^6s^2^6p^2^, Br-3d^10^4s^2^4p^5^ and Cl-3s^2^3p^5^, for which the input files are provided in the pslibrary (version 1.0.0), by implementing the LD1 code included in the QE package. The exchange-correlation (XC) interaction between the valence electrons was described using the Perdew–Burke–Ernzerhof (PBE) functional^49^ within the generalized gradient approximation (GGA) and the Heyd–Scuseria–Ernzerhof (HSE06) hybrid functional.^50^ For virtual atoms X = Br_1−*x*_Cl_*x*_ with increasing Cl content *x* from 0 to 1 with an interval of 0.1, we implemented the virtual.x code provided in the QE package.

For optimization of the CsPbX_3_ unit cell in the cubic phase with a space group of *Pm*3̄*m*, we used the QE package with the kinetic cutoff energies of 60 Ry and 480 Ry for the plane wave basis set for the wave function and electron density and the special *k*-points of (6 × 6 × 6). These computational parameters guarantee the accuracy of the total energy calculation as 0.5 meV per formula unit. Increasing the volume (*V*) of the unit cell evenly from 0.9*V*_0_ to 1.1*V*_0_, where *V*_0_ is the equilibrium volume obtained by optimization, the DFT total energies (*E*) were determined by self-consistent field (SCF) calculations. We fitted the resultant *E*–*V* data to the natural strain equation of state (EOS) for crystalline solid^51^ for each value of Cl content *x*, yielding the bulk modulus and equilibrium lattice constant.

For the electronic band structures and optical properties, we applied the ABINIT package with a kinetic cutoff energy of 40 Ha and special *k*-points of (6 × 6 × 6). The HSE06 hybrid functional^50^ was adopted to describe the XC interaction more precisely, where the portion of exact Hartree–Fock (HF) exchange functional was set to 0.5 for all the Cl content values. The spin–orbit coupling (SOC) effect was considered. We then calculated the effective mass and mobilities of the electron and hole for clarifying the carrier transport properties. The effective mass 
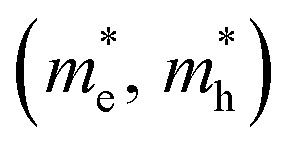
 could be readily obtained by post-processing the resultant band structures using the following formulae,1
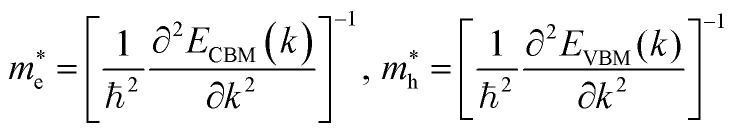
where *E*_CBM_(*k*) and *E*_VBM_(*k*) are the eigen energies as a function of wave number *k* at the conduction band minimum (CBM) and valence band maximum (VBM), respectively. The charge-carrier mobilities (*μ*_e_, *μ*_h_) were calculated with the deformation potential theory^52^ using the following formulae,2
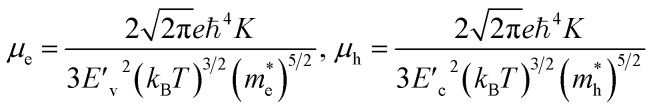
where 
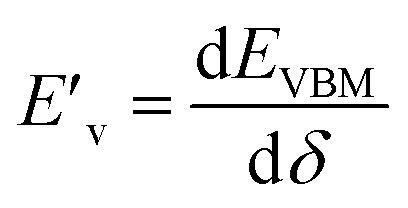
 and 
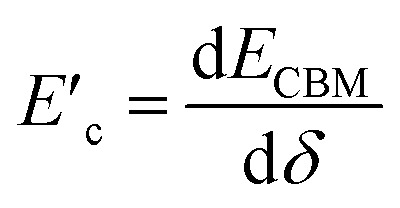
 are the deformation potentials calculated using d*E*_VBM_ and d*E*_CBM_ representing the changes of VBM and CBM energies, and the lattice constant change d*δ* due to a small hydrostatic pressure, and *K* is the bulk modulus.

For the elastic properties including the elastic constants and moduli, we applied the density functional perturbation theory (DFPT) ^53^ using the PBE functional, as implemented in the ABINIT package. For the cubic phase, there are only three independent elastic constants of *C*_11_, *C*_12_ and *C*_44_, from which the bulk modulus *K*, shear modulus *G*, Poisson ratio *ν* and Young’s modulus *E* are given as follows,3
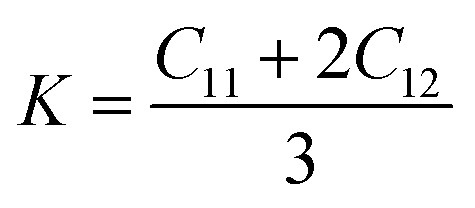
4
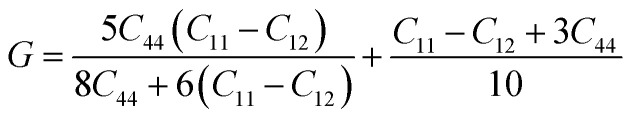
5
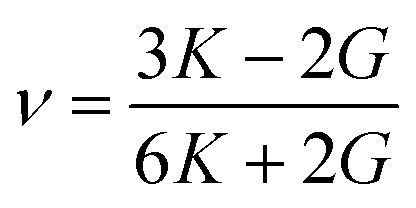

6 *E* = 2*G*(1 + *ν*).

To calculate the frequency dependent dielectric function *ε*(*ω*) = *ε*_1_(*ω*) + *iε*_2_(*ω*), where *ε*_1_(*ω*) and *ε*_2_(*ω*) are the real and imaginary parts of the dielectric function, we solved the Bethe–Salpeter equation including the excitonic effect (BSE-EXC) within the Tamm–Dancoff approximation, as implemented in the ABINIT package. For comparison, we also presented the results obtained within the random phase approximation (RPA) by solving the Kohn–Sham equation (RPA-KS) and GW equation (RPA-GW) with and without a local field effect (LF).^54^ We note that while the KS and GW kernels do not correctly describe the strong excitonic effect within the RPA, the BS kernel is able to capture the most important physics in light absorption. From the dielectric function, the absorption coefficient *α*(*ω*) and reflectivity *R*(*ω*) were calculated using the following formulae,^39,55^7
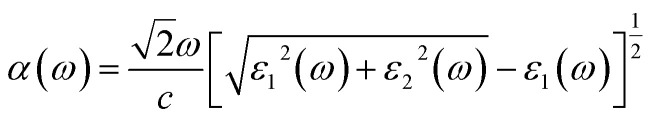
8
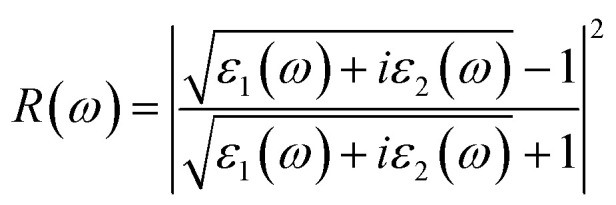


Then, the exciton binding energy *E*_b_ was obtained using the Wannier–Mott hydrogen-like model as follows,^42^9
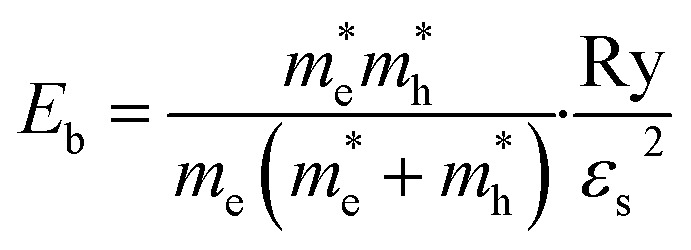
where Ry ≈ 13.56 eV is the Rydberg constant, *m*_e_ is the electron mass, and *ε*_s_ is the static dielectric constant that can be given by *ε*_s_ = *ε*_1_(*ω* → 0).

## 3 Results and discussion

**Fig. 1:**
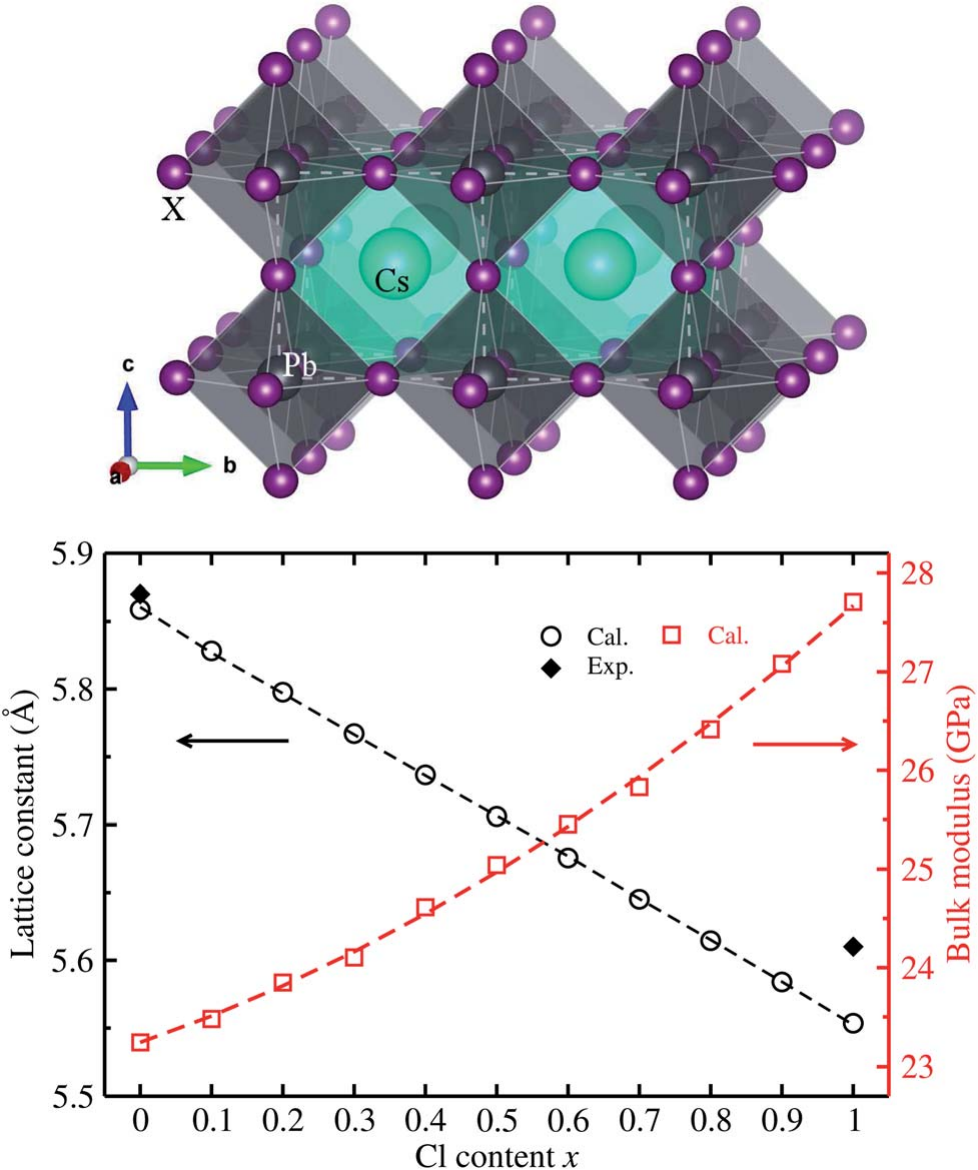
Ball-and-polyhedral view of the crystalline structure of cubic CsPbX_3_ (X = Br_1−*x*_Cl_*x*_) (top panel), where the white-colored dashed lines indicate the unit cell; and calculated lattice constants and bulk moduli are functions of Cl content *x* (bottom panel), where the dashed lines represent the fitted first- and second-order polynomials. Experimental values for lattice constants are from ref. 35.

We first determined the equilibrium lattice constants of Pb-based cesium bromide and chloride perovskite solid solutions of CsPbX_3_ (X = Br_1−*x*_Cl_*x*_) with increasing Cl content *x* from 0 to 1. As described in the method in section 2, the *E*–*V* data was obtained by performing SCF calculations as increasing the unit cell volume evenly from 0.9*V*_0_ to 1.1*V*_0_. The obtained *E*–*V* data was fitted to the natural strain EOS at each value of Cl content *x*, yielding the equilibrium lattice constants and bulk moduli (see Fig. S1† for EOS curves, ESI). Fig. 1 shows the determined lattice constants and bulk moduli as functions of mixing content *x*.

The calculated lattice constants of 5.861 Å for CsPbBr_3_ and 5.552 Å for CsPbCl_3_ were found to be in good agreement with the experimental values of 5.874 and 5.605 Å (ref. 35) with very small relative errors of 0.2% and 0.9%, respectively. This indicates that the pseudopotentials and computational parameters in this work are reasonable. Moreover, it was found that with increasing Cl content *x*, in the solid solutions the equilibrium lattice constant decreases following a linear function of *a*(*x*) = 5.859 − 0.305*x* (Å), which was obtained by fitting the calculated data to the first-order polynomial as shown by the dashed line in Fig. 1. Such linear varying tendency of lattice constants indicates that Vegard’s law can be reproduced well with the VCA approach^44^ for all-inorganic halide perovskite solid solutions. It is worth noting that the lattice constant difference between CsPbBr_3_ and CsPbCl_3_ is relatively small with only a relative difference of 5%, implying the fast halide anion exchange between Br and Cl anions.^22^ On the other hand, the bulk modulus was found to increase following a quadratic function of *K*(*x*) = 23.242 + 2.464*x* + 1.947*x*^2^ (GPa) with increasing Cl content *x*. Such quadratic functions for bulk moduli were also found in other halide perovskite solid solutions within VCA.^39,42,43^ The decrease of lattice constants and increase of bulk moduli are mainly attributed to strengthening of the Pb–X bond, indicating the stability increase in CsPbX_3_ (X = Br_1−*x*_Cl_*x*_) with increasing Cl content *x*.

Using the optimized unit cells, we then calculated the electronic band structures of CsPb(Br_1−*x*_Cl_*x*_)_3_ with increasing Cl content *x*. With a choice of exchange-correlation functional, we remind that the PBE-GGA functional underestimates the band gap while the HSE06 hybrid functional gives band gaps in good agreement with experimental for all-inorganic halide perovskites, and include the SOC effect which severely reduces the band gaps.^56,57^ It is worth noting that SOC can be improved using better core pseudopotentials or explicitly introducing SOC in the Hamiltonian.^58^ In this work, we used the HSE06 hybrid functional with the fixed portion (0.5) of exact HF exchange interaction with and without the SOC effect, to calculate the band structures and band gaps.

**Fig. 2:**
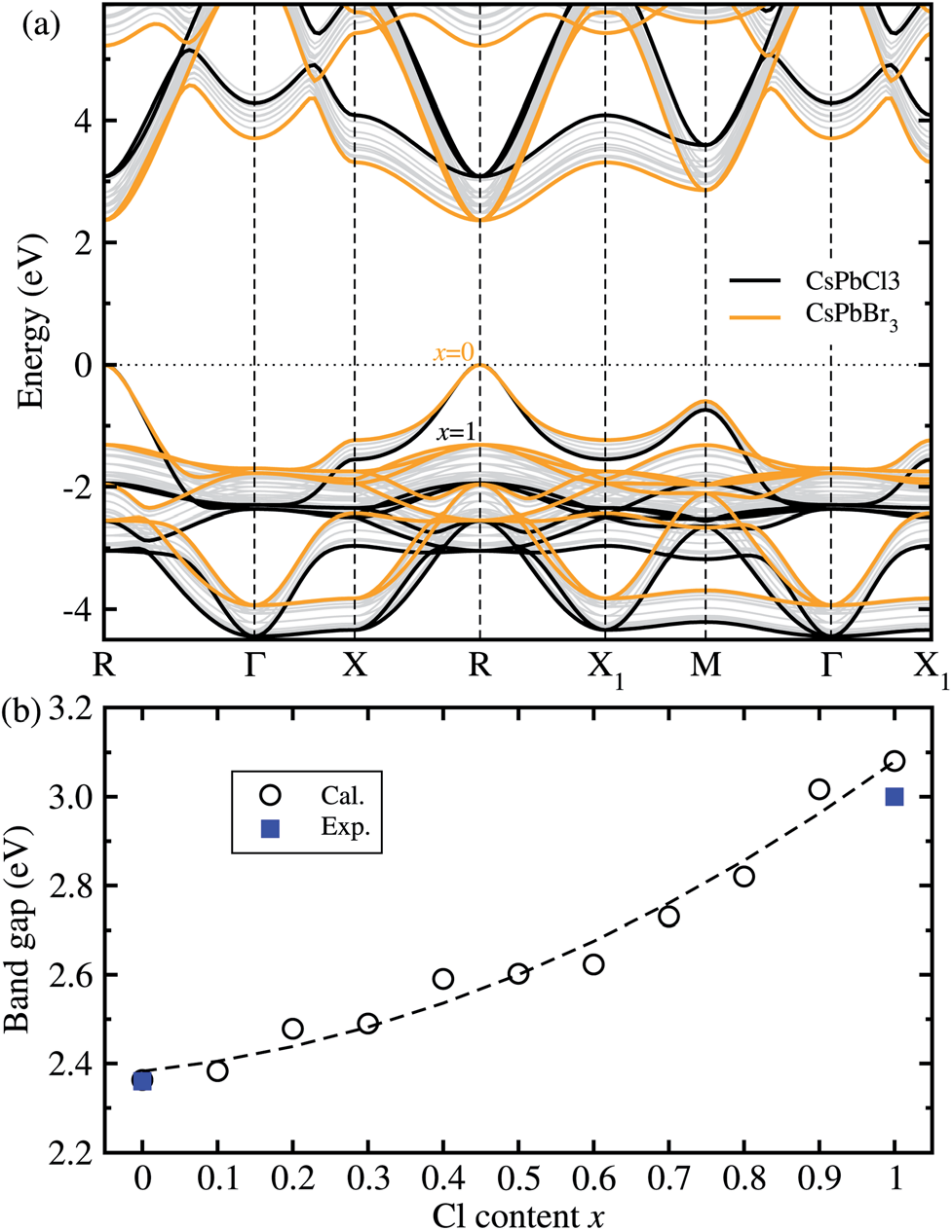
(a) Band structures of cubic CsPbX_3_ (X = Br_1−*x*_Cl_*x*_) with increasing Cl mixing ratio *x* from 0 to 1, calculated using the HSE06 hybrid functional with the fixed portion of exact HF exchange interaction as 0.5, where the valence band maximum is set to zero. (b) Band gaps as a function of mixing ratio, where the dashed line represents the fitting line to the quadratic polynomial. Experimental values are from ref. 15 and ref. 35 for *x* = 0 and 1, respectively.

Fig. 2 shows the band structures and band gaps as a function of mixing ratio calculated with the HSE06 functional without the SOC effect. In Fig. 2(a), one can see a similar dispersion feature overall, and at the direct band gaps at the R point, for all the perovskite solid solutions with the varying mixing ratio values without any anomaly. It was found that with increasing Cl content *x* from 0 to 1 in CsPb(Br_1−*x*_Cl_*x*_)_3_ the VBM moves gradually downwards while the CBM moves upwards at the R point, resulting in the gradual increase of band gap. We note that such a direct transition is more beneficial to absorption and radiation of light than the indirect transition. On the other hand, the HSE06 + SOC method yielded the underestimated band gaps due to a splitting and down-shift of the conduction band known as the Rashba effect (see Fig. S4, ESI†).

For the two extreme compounds at *x* = 0 (CsPbBr_3_) and *x* = 1 (CsPbCl_3_), the direct band gaps were calculated to be 2.36 eV and 3.08 eV, which are in good agreement with the experimental values of 2.36 eV (ref. 15) and 3.03 eV (ref. 35), respectively (see Fig. S5† for comparison between HSE06 and HSE06 + SOC, ESI†). The absolute deviation values with HSE06 are 0 eV and 0.05 eV, indicating that the HSE06 functional is most reliable in reproducing and predicting band gaps of all-inorganic halide perovskites. Fig. 2(b) shows the variation tendency of band gaps as a function of Cl content *x* in the solid solutions. Like other halide perovskite solid solutions, we fitted the calculation data to the quadratic function, resulting in *E*_g_(*x*) = 2.383 + 0.174*x* + 0.522*x*^2^ (eV). In this function, the bowing parameter reflecting the qualitative miscibility between the components of solid solution was found to be 0.52 eV, which is slightly higher than the experimental value of 0.47 eV for CsPb(I_1−*x*_Br_*x*_)_3_.^39^ When compared with the organic–inorganic hybrid solid solutions, this value is smaller than 0.87 eV for MAPb(I_1−*x*_Cl_*x*_)_3_ (ref. 43) but larger than 0.18 eV for MAPb(I_1−*x*_Br_*x*_)_3_.^42^ From the analysis of the bowing parameter, we induce that the compositional disorder and miscibility between CsPbBr_3_ and CsPbI_3_ are reasonable, being better than the organic–inorganic hybrid counterpart. Considering that the composition-dependent band gap indicates the tunability of color emission in a PLED device, one can predict the luminescence peak wavelength of the Br–Cl perovskite solid solution at a certain mixing ratio using this function.

**Fig. 3:**
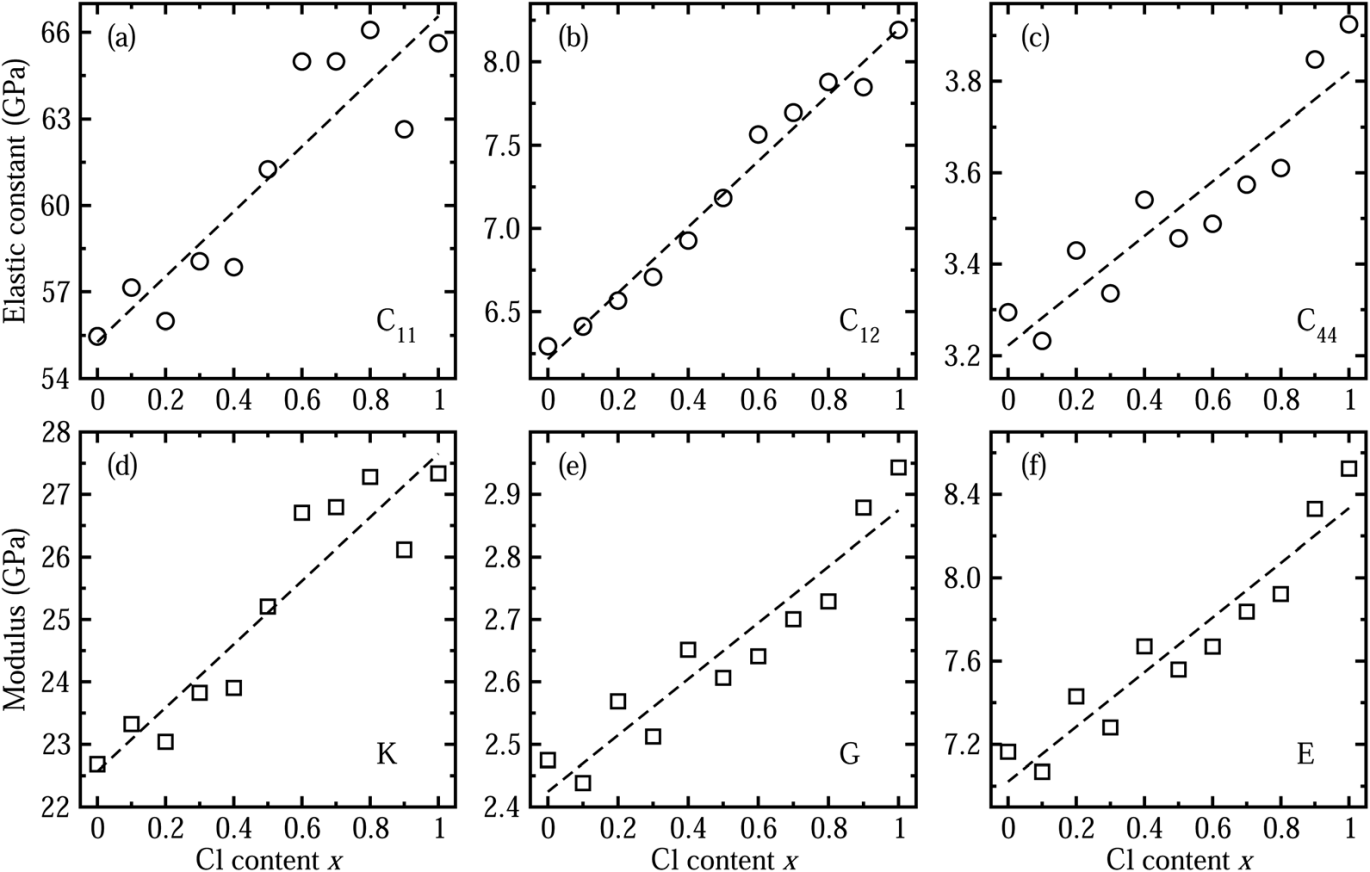
Calculated elastic constants of (a) *C*_11_, (b) *C*_12_, and (c) *C*_44_ of halide perovskite solid solutions of CsPbX_3_ (X = I_1−*x*_Br_*x*_) as functions of Cl content *x*, by applying the DFPT method. (d) Bulk modulus *K*, (e) shear modulus *G*, and (f) Young’s modulus, obtained from the elastic constants. Dashed lines indicate the linear function fit.

Next, we considered the elastic properties including the elastic constants and moduli, since the elastic properties reflect the information on the binding strength, anisotropy and structural stability of the crystal. Fig. 3 shows the elastic constants and moduli obtained by applying the DFPT method using the PBE functional. The three independent components of elastic constant show increasing tendencies with the increase in Cl content *x* along the linear functions of *C*_11_ = 55.275 + 11.283*x* (GPa), *C*_12_ = 6.217 + 1.979*x* (GPa) and *C*_44_ = 3.222 + 0.598*x* (GPa). With increasing Cl content in the solid solution, the bond length between the Pb cation and halide anion becomes shorter as the lattice constant decreases, resulting in enhancement of binding strength and thus structural stability. As such, the bulk modulus *K*, shear modulus *G* and Young’s modulus *E*, which are estimated from the elastic constants, were also found to linearly increase following *K* = 22.570 + 5.081*x* (GPa), *G* = 2.424 + 0.450*x* (GPa), and *E* = 7.022 + 1.312*x* (GPa), respectively. We confirmed that the calculated bulk moduli for the two end compounds are in good agreement with the previous theoretical work.^55^ We also estimated the anisotropy factor as *A* = 2*C*_44_/(*C*_11_ − *C*_12_), which was found to be between 0.12 and 0.14 for CsPb(Br_1−*x*_Cl_*x*_)_3_ with *x* = 0–1, with severe deviation from 1 for an isotropic crystal.

For optoelectronic applications like solar cells and LEDs, the effective mass and mobilities of the electron and hole are of great importance, and are prerequisite factors for perovskites to become the active layers in any device.^55,59,60^ The effective mass of charge carriers was obtained from the detailed band structures (see Fig. 2) calculated with the HSE06 functional by applying the parabolic approximation around the R point of the conduction and valence bands using eqn (1). To evaluate the carrier mobilities using eqn (2), we recalculated the band structures by varying the lattice constant from 99.8% to 100.2% with an interval of 0.1% and then obtained the deformation potentials as the gradients of the CBM and VBM energies with respect to the lattice constant change at each value of Cl content *x*.

**Fig. 4:**
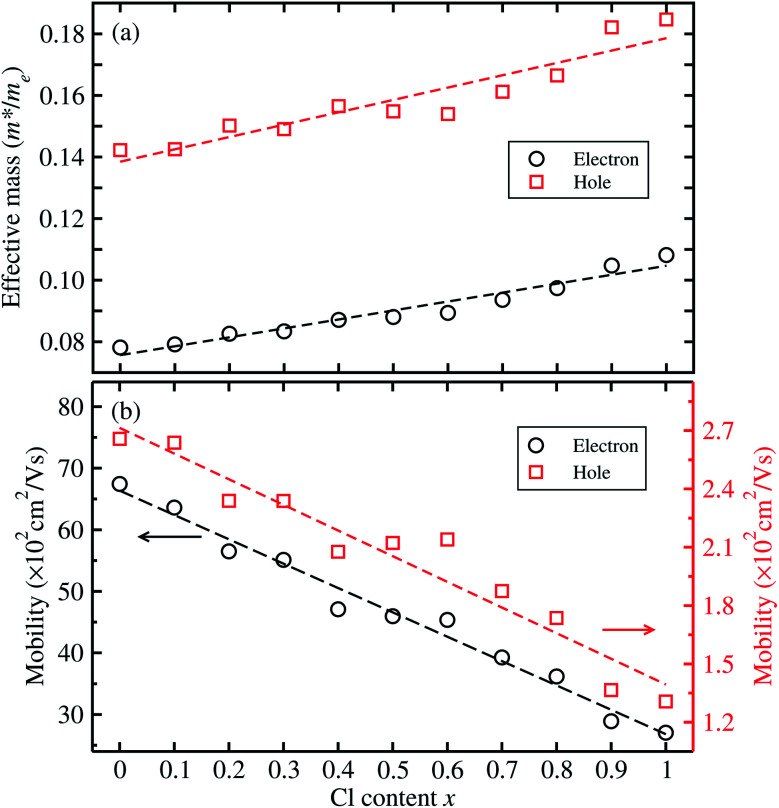
(a) Effective mass of electron and hole calculated from the band structures with the HSE06 functional, and (b) electron and hole mobilities calculated from the effective mass, bulk moduli and deformation potentials, as a function of Cl content *x* in solid solutions of CsPb(Br_1−*x*_Cl_*x*_)_3_. Dashed lines indicate lines of fit.

Fig. 4 shows the calculated effective mass and mobilities of the electron and hole as functions of Cl content *x* in solid solutions of CsPbX_3_. For the two extreme compounds, the effective mass of electron and hole were calculated to be 
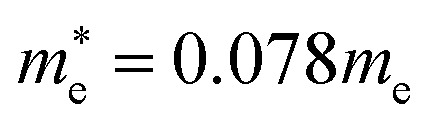
 and 
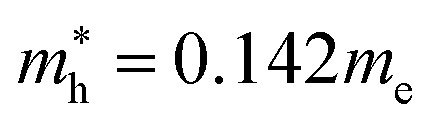
 for CsPbBr_3_ and 
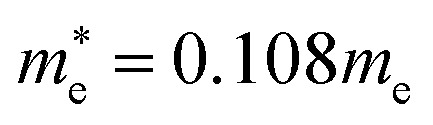
 and 
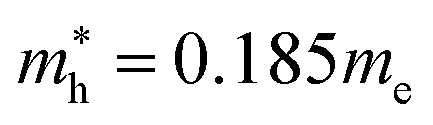
 for CsPbCl_3_, which are clearly smaller than 
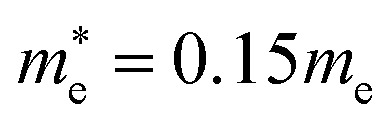
 and 
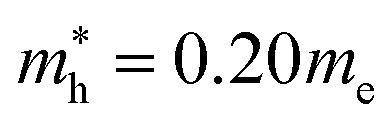
 for CsPbI_3_.^55^ When compared with the organic–inorganic hybrid counterparts of MAPbBr_3_ and MAPbCl_3_, where MA is the methylammonium (CH_3_NH_3_) cation, CsPbBr_3_ has a much lighter effective mass than 
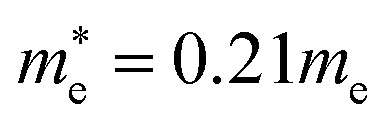
 and 
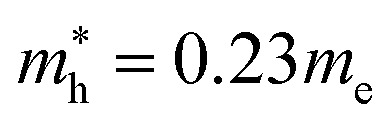
 for MAPbBr_3_ (ref. 42) and CsPbCl_3_ also has a lower effective mass than 
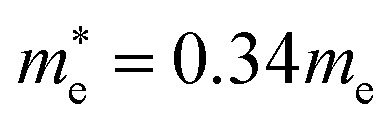
 and 
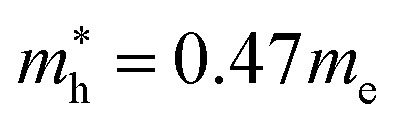
 for MAPbCl_3_.^43^ These indicate that both CsPbBr_3_ and CsPbCl_3_ have better charge carrier transport properties than CsPbI_3_, MAPbBr_3_ and MAPbCl_3_. Since the effective mass in CsPbCl_3_ is larger than in CsPbBr_3_, it can be expected that mixing bromide with chloride in a perovskite, forming solid solutions, will induce an increase in effective mass but will still be lower than CsPbCl_3_. In fact, it was revealed that both the electron and hole effective mass of solid solutions increases with increasing Cl content *x* along the linear functions of 
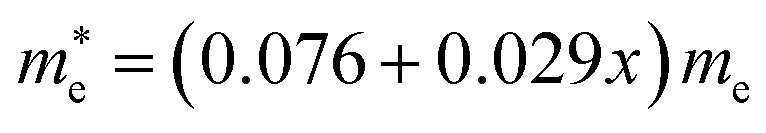
 and 
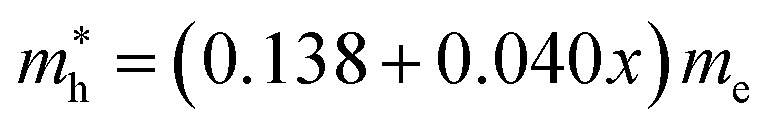
, respectively. We note that the electrons are almost twice as light as the holes in the active layer of the device after their generation by photon excitation or charge injection in the CsPb(Br_1−*x*_Cl_*x*_)_3_-based PSCs or PLEDs. In accordance with the change tendencies of effective mass, the charge carrier mobilities were found to decrease with increasing Cl content along the linear functions of *μ*_e_ = (66.360 − 39.548*x*) × 10^2^ cm^2^ V^−1^ s^−1^ and *μ*_h_ = (2.714 − 1.320*x*) × 10^2^ cm^2^ V^−1^ s^−1^. Again, it was found that the electron mobilities are much larger than the hole mobilities.

**Fig. 5:**
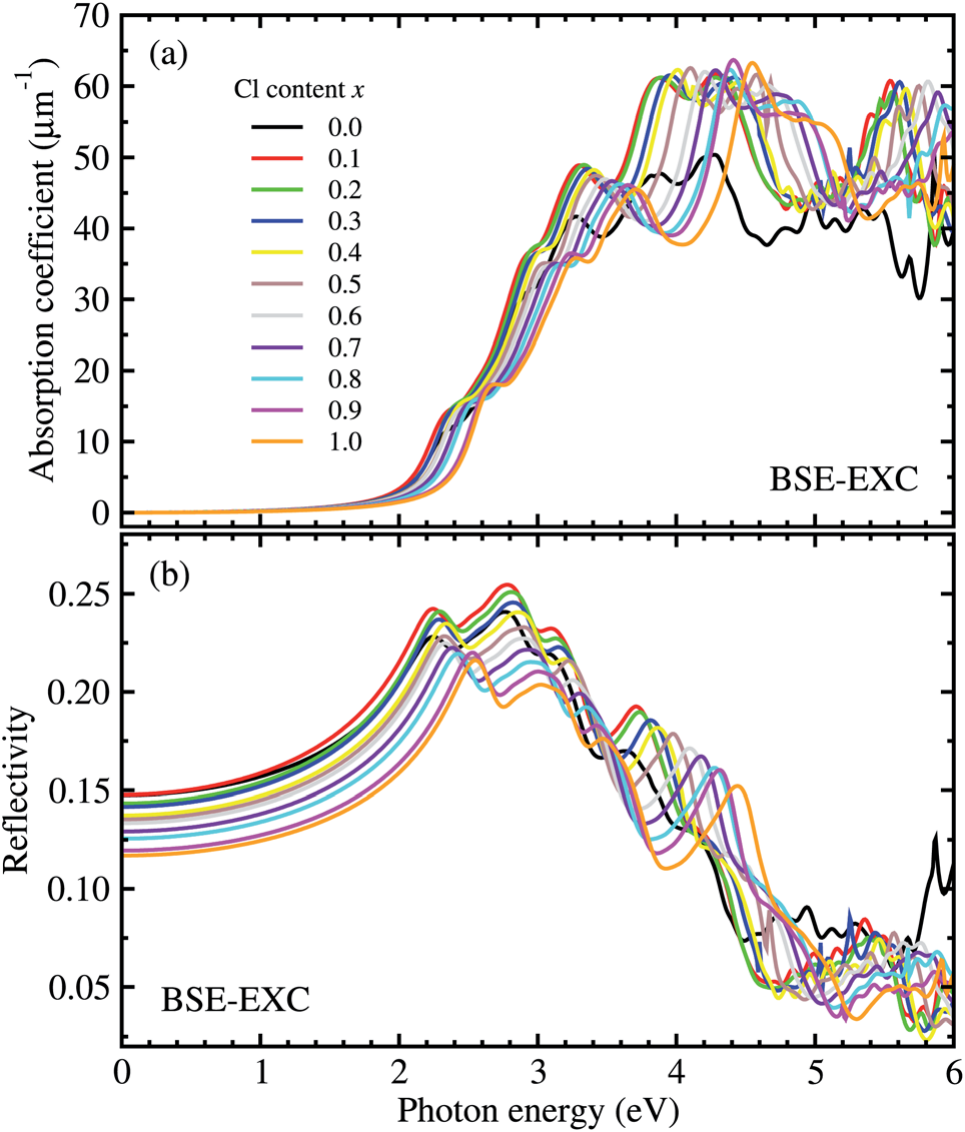
(a) The photo-absorption coefficient and (b) reflectivity of perovskite solid solutions of CsPb(Br_1−*x*_Cl_*x*_)_3_ with Cl content *x* = 0–1 as functions of photon energy, are obtained from the frequency dependent dielectric functions calculated by solving the Bethe–Salpeter equation (BSE) including the excitonic effect (EXC).

For optical properties, we calculated the frequency dependent dielectric function of solid solutions of CsPbX_3_ (X = Br_1−*x*_Cl_*x*_). To this end, we solved the Bethe–Salpeter equation (BSE) incorporating the excitonic effect (EXC), *i.e.*, interactions between electron and hole. Due to the cubic symmetry of CsPbX_3_, we averaged the identical *xx*, *yy*, and *zz* components of the dielectric tensor (see Fig. S2† for real and imaginary parts of the dielectric functions, ESI). For comparison, the frequency dependent dielectric constants were also calculated using different levels of theory such as RPA-KS and RPA-GW (see Fig. S3, ESI†). The photo-absorption coefficients and reflectivity were obtained from these dielectric functions using eqn (7) and (8). Fig. 5 shows the calculated photo-absorption coefficients and reflectivity of solid solutions of CsPb(Br_1−*x*_Cl_*x*_)_3_ with increasing Cl content *x* from 0 to 1 with an interval of 0.1. The absorption onset was observed to be shifted to the ultraviolet region when increasing the Cl content, which coincides with the tendency of band gap change as discussed above.

**Fig. 6:**
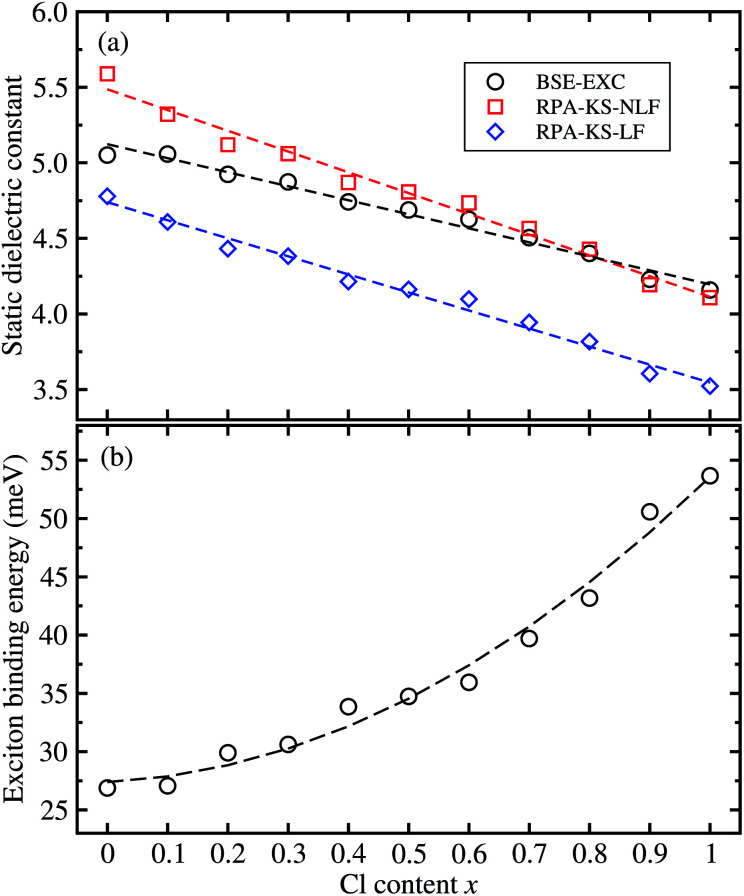
(a) Static dielectric constants as a function of the Cl content *x* in solid solutions of CsPb(Br_1−*x*_Cl_*x*_)_3_, calculated by solving the Bethe–Salpeter equation including the excitonic effect (BS-EXC) or by density functional perturbation theory within the random phase approximation in the Kohn–Sham approach (RPA-KS) without the local field effect (NLF) and with LF. (b) The exciton binding energy is obtained from the BS-EXC result.

From the real part of the dielectric function calculated by the BS-EXC approach or RPA-KS-NLF and RPA-KS-LF approaches, we extracted the static dielectric constant as *ε*_s_ = *ε*_1_(*ω* → 0). For CsPbBr_3_ and CsPbCl_3_, the static dielectric constants were determined to be 5.05 and 4.16 from the BS-EXC results, which are slightly smaller than the previous theoretical values of 4.20 and 3.66 obtained with PBE + SOC.^55^ These values are also slightly smaller than those of the organic–inorganic hybrid counterparts of MAPbBr_3_ (3.80) ^42^ and MAPbCl_3_ (3.11).^43^ When increasing the Cl content *x*, we found the increasing tendency of the static dielectric constants along the linear function of *ε* = 5.124 − 0.928*x*, as shown in Fig. 6(a). In this figure, we also show the DFPT calculation results for comparison. It was found that the RPA-KS-NLF approach gave slightly larger or similar values with a linear function of *ε* = 5.487 − 1.375*x*, whereas the RPA-KS approach including the local field effect, yielded much smaller values with a linear function of *ε* = 4.740 − 1.194*x*.

Finally, we determined the exciton binding energy *E*_b_ from the calculated effective mass and static dielectric constants with BS-EXC using eqn (9). The exciton binding energy implies the stability of the exciton comprised of photo-generated electron and hole; a lower value means faster carrier dissociation, being favourable for the solar cell or LED application. For CsPbBr_3_, *E*_b_ was found to be 26.9 meV in good agreement with the experimental value of 26 meV.^61^ The calculated value of 53.7 meV for CsPbCl_3_ is comparable with previous experimental and theoretical values.^36,55,61^ These values can be thought of as relatively small, indicating the weak binding in the exciton, *i.e.*, Mott–Wannier exciton, which is beneficial for the fast dissociation of the exciton into free charge carriers. As shown in Fig. 6(b), the calculated values of solid solutions of CsPb(Br_1−*x*_Cl_*x*_)_3_ show an increasing tendency with increasing Cl content *x*, according to the quadratic function of *E*_b_ = 27.389 + 2.473*x* + 23.711*x*^2^ (meV). Such increasing tendency is reasonable because the effective mass of the electron and hole become smaller and the static dielectric constants decrease with increasing Cl content. In Table 1, we summarize the fitted functions for the calculated data in comparison with other halide perovskite solid solutions, which will be helpful for material designers and engineers.

**Table 1:** Fitted functions obtained by regressing data calculated within VCA to the first- or second-order polynomials in comparison with the previous data for other halide perovskite solid solutions of CsPb(I_1−*x*_Br_*x*_)_3_,^39^ MAPb(I_1−*x*_Br_*x*_)_3_,^42^ and MAPb(I_1−*x*_Cl_*x*_)_3_ (ref. 43)

	CsPb(Br_1−*x*_Cl_*x*_)_3_	CsPb(I_1−*x*_Br_*x*_)_3_ (ref. 39)	MAPb(I_1−*x*_Br_*x*_)_3_ (ref. 42)	MAPb(I_1−*x*_Cl_*x*_)_3_ (ref. 43)
Lattice constant (Å)	5.859 − 0.305*x*	6.242 − 0.378*x*	6.420 − 0.333*x*	6.310 − 0.716*x*
Bulk modulus (GPa)	23.242 + 2.464*x* + 1.947*x*^2^			10.627 + 5.492*x*
Band gap, HSE06 (eV)	2.383 + 0.174*x* + 0.522*x*^2^	2.245 + 0.067*x* + 0.071*x*^2^		
PBE or PBEsol		1.759 + 0.012*x* + 0.032*x*^2^	1.542 + 0.374*x* + 0.185*x*^2^	1.521 + 0.269*x* + 0.873*x*^2^
Electron effective mass (*m*_e_)	0.076 + 0.029*x*			0.199 + 0.140*x*
Hole effective mass (*m*_e_)	0.138 + 0.040*x*			0.231 + 0.203*x*
Static dielectric constant	5.124 − 0.928*x*	5.077 − 0.451*x*	5.230 − 1.430*x*	5.698 − 2.593*x*
Exciton binding energy (meV)	27.389 + 2.473*x* + 23.711*x*^2^		45 + 57*x*	55 − 36*x* + 25.3*x*^2^
Elastic constant *C*_11_ (GPa)	55.275 + 11.283*x*			
Elastic constant *C*_12_ (GPa)	6.217 + 1.979*x*			
Elastic constant *C*_44_ (GPa)	3.222 + 0.598*x*			
Electron mobility (× 10^2^ cm^2^ V^−1^ s^−1^)	66.360 − 39.548*x*			
Hole mobility (× 10^2^ cm^2^ V^−1^ s^−1^)	2.714 − 1.320*x*			

## 4 Conclusions

In this work we have performed first-principles calculations within the virtual crystal approximation to systematically investigate the structural, elastic, electronic and optical properties of all-inorganic halide perovskite solid solutions of CsPb(Br_1−*x*_Cl_*x*_)_3_ in the cubic phase with varying Cl content *x*. Confirming that the calculated lattice constants of the two extreme compounds were in good agreement with the experimental values, we found that the equilibrium lattice constants of the solid solutions increased along the linear function of *a*(*x*) = 5.859 − 0.305*x* (Å) with increasing Cl content *x*, indicating the satisfaction of Vegard’s law and reliability of the pseudopotentials of the virtual atoms. Our calculations revealed that the band gaps calculated with HSE06 increased quadratically according to the function of *E*_g_(*x*) = 2.38 + 0.17*x* + 0.52*x*^2^, predicting the luminescence peak wavelength of the solid solution at a certain mixing ratio. We found that the elastic constants and moduli exhibited a monotonic increasing tendency with the increase of Cl content, implying that the binding strength and structural stability can be enhanced upon mixing bromide with chloride. For the charge-carrier transport properties, we calculated the effective mass of electron and hole, showing the linear increasing tendencies of 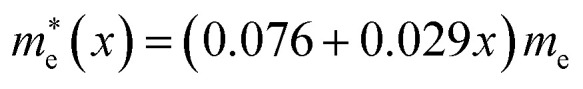 and 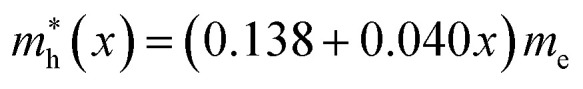 and thereby the linear decreasing tendencies for mobilities. We then computed the frequency dependent dielectric functions with BS-EXC and the photo-absorption coefficients, finding that the absorption picks were shifted to the ultraviolet region with increasing Cl content. Finally, we found that the static dielectric constants decreased along the linear function of *ε*(*x*) = 5.124 − 0.928*x*, while the exciton binding energies increased according to the quadratic function of *E*_b_ = 27.39 + 2.47*x* + 23.71*x*^2^ (meV), revealing the weak binding strength of exciton and its fast dissociation into free carriers. We believe that this work can provide useful guidance for developing active materials based on halide perovskites for PLEDs.

## Author contributions

Un-Hyok Ko and Chol-Jun Yu developed the original project, performed the calculations and drafted the first manuscript. Jun-Hyok Ri, Jong-Hyok Jang, Chol-Hyok Ri and Un-Gi Jong assisted with the DFT calculations and the post-processing of calculation results, and contributed to useful discussions. Chol-Jun Yu supervised the work. All authors reviewed the manuscript.

## Conflicts of interest

There are no conflicts to declare.

## Supplementary Material

RA-012-D2RA01084D-s001
