# Supplementary material for: First-principles study on the elastic, electronic and optical properties of all-inorganic halide perovskite solid solutions of CsPb(Br1−xClx)3 within the virtual crystal approximation
Source: RSC Adv. 2022 Mar 29;12(16):9755–62. doi: 10.1039/d2ra01084d (PMC8961602; doi:10.1039/d2ra01084d)
Supplement: RA-012-D2RA01084D-s001 [file RA-012-D2RA01084D-s001.pdf]

Electronic Supplementary Material (ESI) for RSC Advances.
This journal is © The Royal Society of Chemistry 2022

Supporting Information – First-principles study on elastic, electronic and optical properties of all-inorganic halide perovskite solid solutions $CsPb(Br_{1-x}Cl_x)_3$ within virtual crystal approximation

Un-Hyok Ko, Jun-Hyok Ri, Jong-Hyok Jang, Chol-Hyok Ri, Un-Gi Jong, Chol-Jun Yu*

Chair of Computational Materials Design (CMD), Faculty of Materials Science, Kim Il Sung University, Ryongnam-Dong, Taesong District, Pyongyang, PO Box 76, Democratic People's Republic of Korea

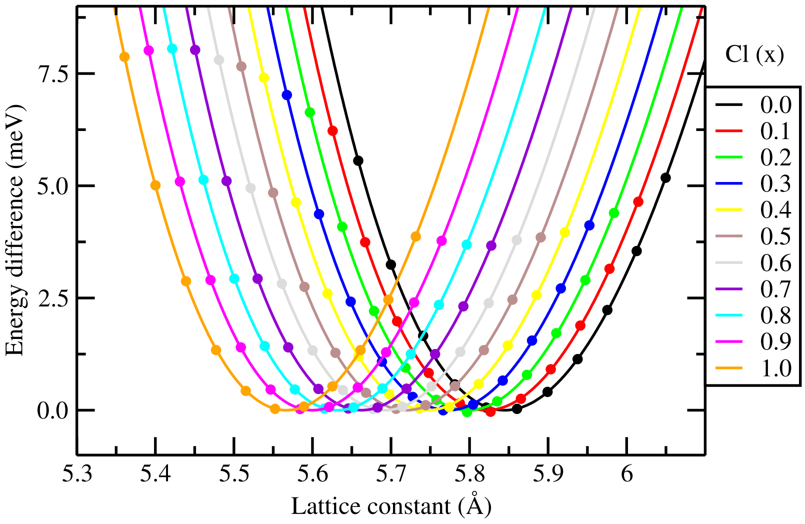

Figure S1. Energy–lattice constant curves obtained by fitting the calculation data into the natural strain equation of state (EOS) for crystalline solid solutions $CsPb(Br_{1-x}Cl_x)_3$ (x = [0, 1], interval 0.1).

*Corresponding author: Chol-Jun Yu, Email: cj.yu@ryongnamsan.edu.kp

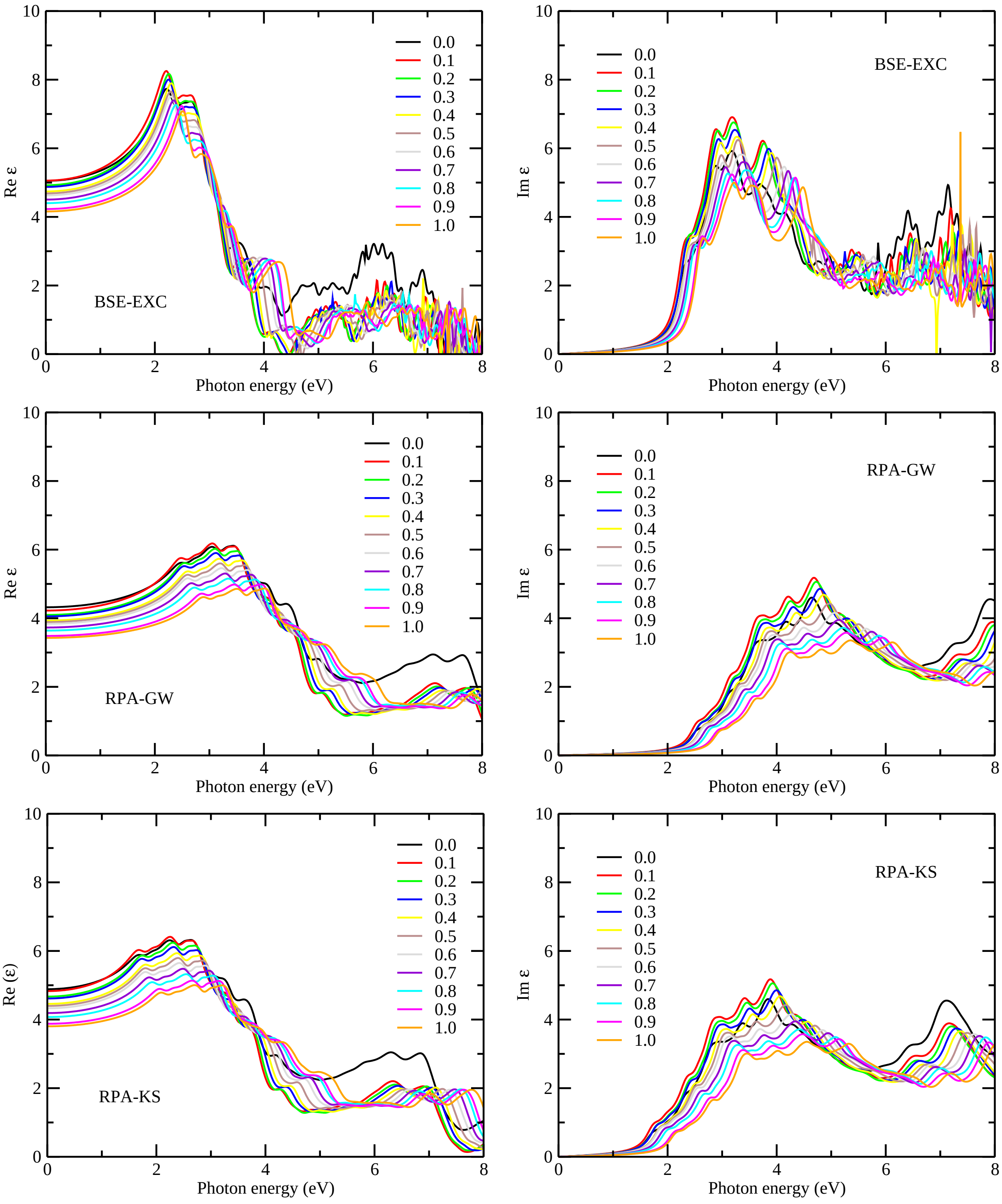

Figure S2. Frequency dependent dielectric constants of $CsPb(Br_{1-x}Cl_x)_3$ (x = [0, 1], interval 0.1), obtained by solving the Bethe-Salpeter equation with excitonic effect (BSE-EXC), *GW* equation within random phase approximation (RPA-*GW*) and Kohn-Sham equation with RPA (RPA-KS).

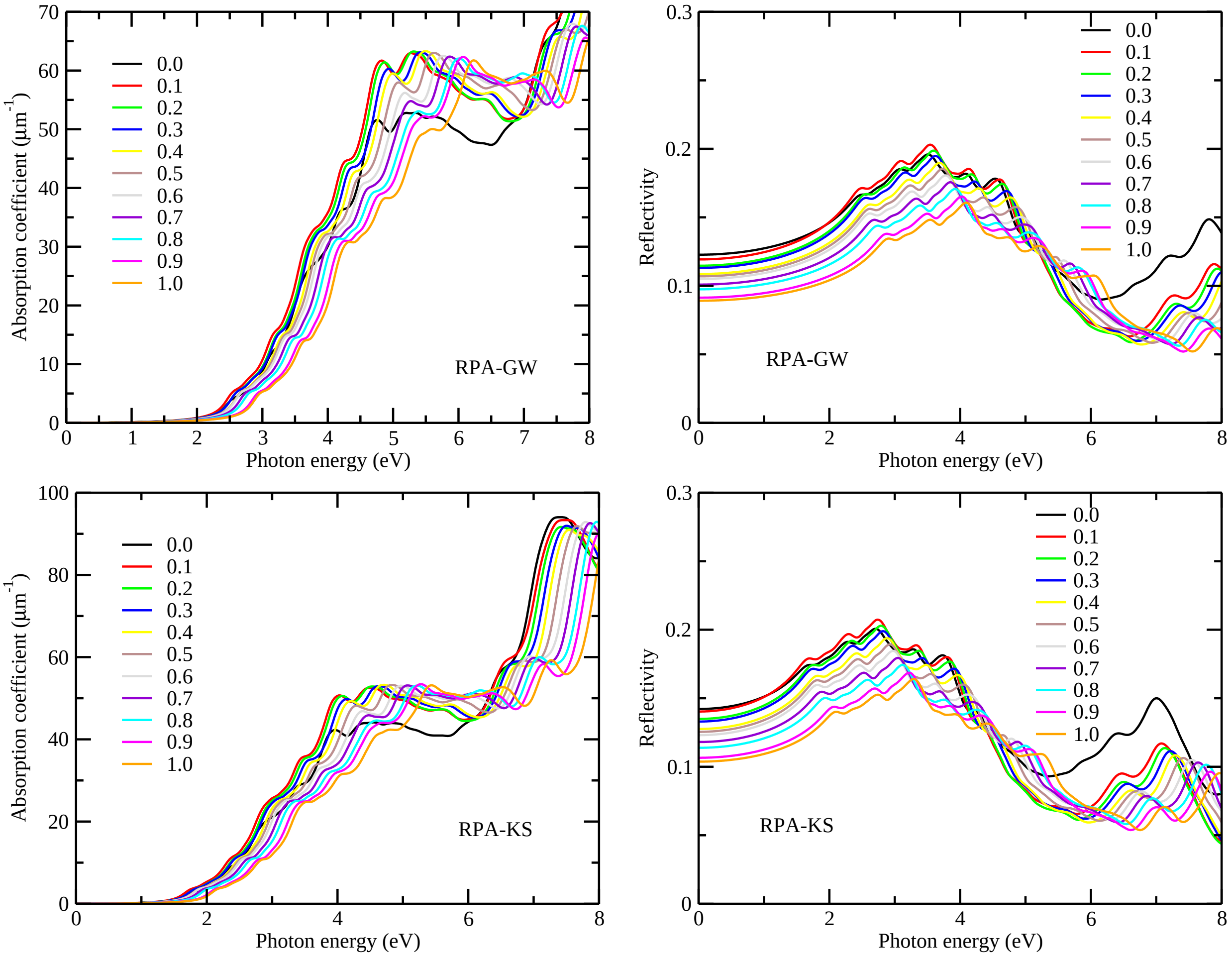

Figure S3. Photo-absorption coefficient (left panel) and reflectivity (right panel) of $CsPb(Br_{1-x}Cl_x)_3$ as a function of photon energy, obtained by using RPA-*GW* and RPA-KS approaches.

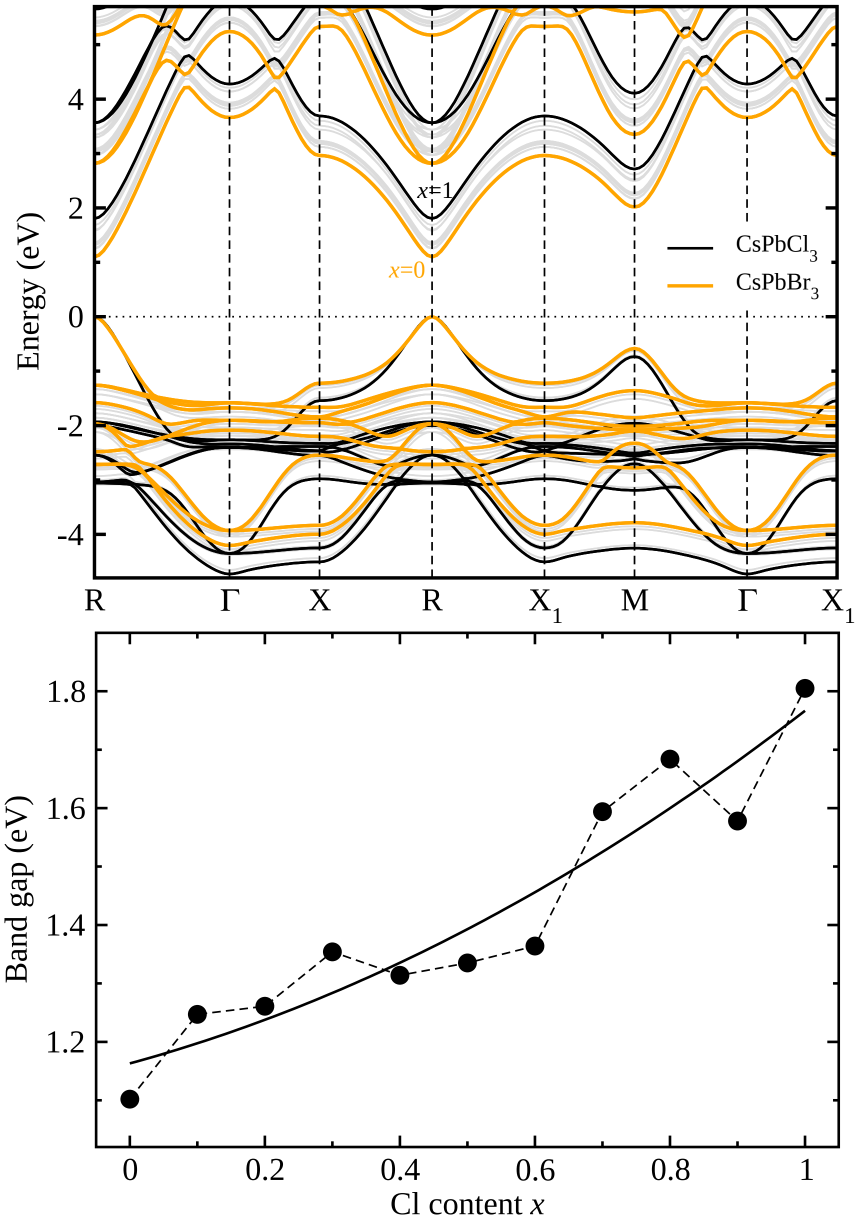

Figure S4. Band structures of cubic $CsPbX_3$ (X = $Br_{1-x}Cl_x$) (top) and band gaps (bottom) as increasing the Cl content x from 0 to 1, calculated with HSE06+SOC method. The band gap increases along the quadratic function of $E_g(x) = 1.163 + 0.316x + 0.288x^2$ (eV).

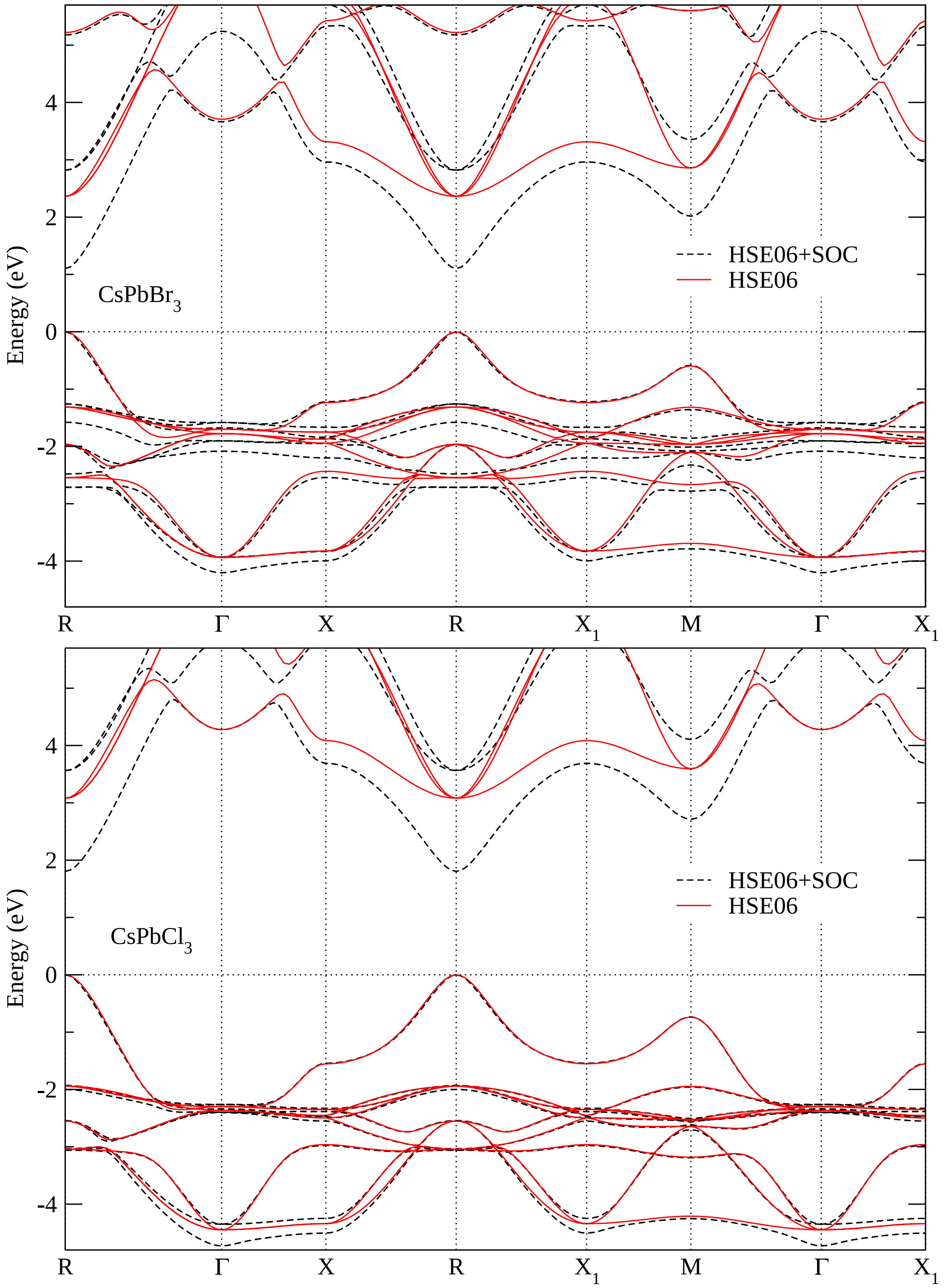

Figure S5. Comparison of band structures calculated with HSE06 (red solid line) and HSE06+SOC (black dashed line) for $CsPbBr_3$ (top) and $CsPbCl_3$ (bottom).
